# Risk factor comparison in young patients presenting with acute coronary syndrome with atherosclerotic coronary artery disease vs. angiographically normal coronaries

**DOI:** 10.7150/ijms.60869

**Published:** 2021-08-21

**Authors:** Sarah Jamil, Gohar Jamil, Hanaa Mesameh, Anwer Qureshi, Juma AlKaabi, Charu Sharma, Faisal Aziz, Ali Rashed Al-Shamsi, Javed Yasin

**Affiliations:** 1 Internal Medicine, Tawam Hospital, Al Ain, United Arab Emirates.; 2 Department of Cardiology, Tawam Hospital, Al Ain, United Arab Emirates.; 3 Department of Internal Medicine, College of Medicine and Health Sciences, United Arab Emirates University, Al Ain, United Arab Emirates.; 4 Nursing, Tawam Hospital, Al Ain, United Arab Emirates.; 5 Geisinger Medical Center, Danville, PA, USA.; 6 Medical University of Graz, Graz, Austria.

**Keywords:** young, acute coronary syndrome (ACS), coronary artery disease (CAD), coronary angiography, risk factors

## Abstract

**Background:** Acute myocardial infarction is a relatively rare phenomenon in the young population. The incidence has nevertheless increased from years past, likely due to the presence of multiple risk factors from an increasingly younger age. Regardless of whether they have atherosclerotic coronary artery disease or normal coronary angiogram, young patients with risk factors for coronary artery disease (CAD), chest pain, and positive troponin, are initially treated in a similar fashion. Our goal was to shed light on whether risk factors between these two groups differ to help guide physicians in clinically determining whether or not an atherosclerotic cardiovascular event has occurred, as well as to potentially identify young patients at risk of acute coronary syndrome (ACS) despite normal coronary arteries.

**Methods**: A retrospective cross sectional study was undertaken over an 8 year period at Tawam Hospital. 576 patients aged 50 or under who underwent coronary angiography were selected for the study. Medical records were analyzed for the patient's demographics and CAD risk factor profile, including the following variables: family history of CAD, smoking status, Body Mass Index category, lipid profile, and diagnosis of hyperlipidemia, diabetes, or hypertension. Details of the coronary angiogram were also reviewed.

**Results:** Statistically significant outcomes included a higher prevalence of diabetes, hyperlipidemia, and smoking history in patients with CAD compared to the patients with normal coronary angiogram. Diabetes was one of the strongest risk factors in CAD patients, with an odds ratio of 1.98 (p= 0.011), followed by hyperlipidemia at 1.85 (p= 0.021). Smoking history had an odds ratio of 2.93 (p <0.001).

**Conclusion:** Risk factors were present in both groups, but significantly more in the CAD group. No particular risk factor stood out for the development of ACS in those with normal coronary arteries, other than mean BMI being slightly higher in this group. Based on our analysis, no single variable can accurately predict the risk for ACS in normal coronaries. To our knowledge, few studies have been done in the young population with angiographically normal coronary arteries to determine possible risk factors for development of ACS. Further research needs to be done to determine whether the risk factors that were common amongst both groups are coincidental, or a cause of ACS in those with normal coronary arteries.

## Introduction

The incidence of myocardial infarction (MI) among the younger population (under the age of 45 years) is around 2-10% 1. As age increases, the presence of risk factors in CAD patients increases. In 15-19-year-olds, only 2% of males and 0% of females had major risk factors, whereas in 30-34 year age group, this increased to 20% and 8%, respectively 2-3. These percentages may be perceived as infrequent, but when taking absolute numbers into account, it is indicated that the occurrence does not remain uncommon. In Brazil, 4549 patients below 45 years of age were hospitalized with acute myocardial infarction (AMI) in 2000 4. In the Framingham Heart Study, the 10 year incidence of an MI was 12.9/1000 in 30 to 34 year old men and 5.2/1000 in 35 to 44 year old women 5. The incidence has risen in recent years, in part due to an increasing prevalence of risk factors for cardiovascular disease in the younger population, undermining the protective effect of young age. As a consequence, this is of rising clinical interest because of the potential of premature death and long-term disability 6-7.

The existence of ACS despite the presence of angiographically normal coronary arteries was recognized many years ago, however the etiology of the condition is still under speculation and not much information is available in current literature 8-10. A single explanation for the occurrence of AMI in the presence of apparently normal coronary arteries is absent, but various mechanisms have been proposed 8. One study evaluated the diagnostic value of cardiac MRI (CMRI) in ACS patients with normal coronary arteries and found that a majority had no late gadolinium enhancement (LGE) on CMRI 11. We seek to discern if there is any association between the angiographic status of young patient's coronary arteries and their cardiovascular risk factors. The purpose of our study was knowing the difference in risk factors between the two groups (young patients with atherosclerotic coronary arteries versus those with no atherosclerosis), which may be of importance when risk stratifying, prescribing medication, and considering different modes of treatment in the future, especially for those with normal coronary arteries. We sought to answer the question: is it possible to differentiate which patients have normal coronary arteries based on a certain risk factor profile?

## Material and Methods

A retrospective cross sectional study was undertaken, the objective being to compare the risk factors for coronary artery disease present in those with atherosclerotic coronary artery disease versus the risk factors in those with angiographically unremarkable coronary arteries, and identifying any discernible differences.

There were a total of 593 patients selected for the study over an 8 year period at Tawam Hospital, a government tertiary care hospital (from 2008 to 2015). Approval was obtained from the Ethics Committee. All patients who underwent coronary angiography and were admitted with the presumptive diagnosis of ACS, aged 50 or under, were included. Patients who were diagnosed with ACS but did not undergo angiography were excluded. Medical records were analyzed for the patient's CAD risk factor profile including variables such as: age, sex, ethnicity, family history of coronary heart disease, smoking status, BMI (Body Mass Index) category, total cholesterol levels (as well as separate categories for LDL, HDL, and TG), and diagnosis of hyperlipidemia, diabetes, or hypertension. Coronary angiography was done by interventional cardiologists at Tawam Hospital on the Siemens system. Presence or absence of CAD on angiogram was used to allocate participants into the two groups. Details of the coronary angiogram, laboratory investigations, and metabolic and cardiac biomarkers were also reviewed. For each risk factor patients were further subcategorized according to the condition of their coronary arteries (atherosclerotic or normal), and the results evaluated to see whether the analyzed risk factor was comparable, less prevalent, or more prevalent in those with atherosclerosis or not.

### Statistical Analysis

Data were inspected for outliers for continuous variables and implausible values for categorical variables. The outliers were visually inspected and verified in the patient records and replaced accordingly. The outliers that were found to be correct in the patient records were retained in the data. Both outliers and implausible entries were verified in the database and corrected if required, in Stata 15.1. We assessed the normality of all continuous variables included in the study using both graphical (qqplot) and statistical methods (Kurtosis, Skewness, Shapiro-Wilk test). We found no evidence of significant deviation from the normality. Risk factors were tabulated overall and by normal versus atherosclerotic disease as mean ± standard deviation (SD) for quantitative variables and frequencies (%) for qualitative variables as appropriate. Two sample t-tests were performed to compare quantitative variables between groups and Chi-square tests were performed to compare qualitative variables. Simple and multiple logistic regression analysis were performed to estimate crude and adjusted odds ratios (OR), 95% confidence intervals (CI) and p-values of atherosclerotic CAD with selected risk factors. The p-value <0.05 was selected to determine the statistical significance of risk factors with atherosclerotic CAD. Since it was a retrospective analysis, no power calculation was carried out prior to the study and all patients fulfilling the inclusion criteria were included in the analysis. However, we have calculated the post-hoc power of outcome with each significant risk factor. In this regard, we have chosen the average odds ratio of 2, 95% CI, and alpha error of 5% per each significant risk identified in this study. According to these assumptions, the study has a power of more than 90% to assess the association of each risk factor with the outcome.

## Results

Table 1 shows summary statistics of risk factors overall and by atherosclerotic CAD. Overall, the mean age of participants was 37.9 ± 5.3 years. The majority of participants were males (87.7%) and migrants from sub-continent (53.7%). One-fourth of participants had diabetes, hyperlipidemia and obesity each, while 34.3% had hypertension. See Table 1 for details.

Diabetes was one of the strongest risk factors encountered, with 110 patients (28.3%) with abnormal coronary arteries having diabetes (vs only 20 (16.7%) with normal coronary arteries (p = 0.010).When comparing coronary anatomy amongst different age ranges as a risk factor in our cohort, it becomes evident that increasing age was related to an increase in the risk of atherosclerotic CAD (p < 0.001). Even in those with normal coronary anatomy, increasing age was associated with a higher number of patients' presentation.

Males had a significantly higher likelihood of atherosclerosis (p <0.001), with only 85 males having normal coronary arteries. Only 6.9% (32) of patients with CAD were female, an overwhelming 93.1% (435) were male. More females had normal coronary arteries (41) than abnormal arteries (32).

Among different ethnicities, those from the subcontinent were more likely to have atherosclerotic CAD (246, 61.5%) as compared to their Emirati (79, 19.7%) and Arab (66, 16.5%) counterparts, and other nationalities (9, 2.3%). Among those who were found to have angiographically normal coronary arteries, the variation in distribution between different nationalities was less marked. 31.7% (38) were Emirati, 30.0% (36) were Arab, 27.5% (33) were from the subcontinent, and 10.8% (13) belonged to other nationalities.

A history of smoking was also pertinent in those with atherosclerotic CAD. 240 (63.3%) patients who were found to have CAD were smokers, while only 43 (37.1%) patients with normal coronary arteries were smokers (p <0.001).

Mean BMI was significantly higher in those with normal (29.7) vs abnormal (27.5) coronary arteries (p = 0.008).

There was no difference in cholesterol levels among the two groups, save for TG (p = 0.047). Hyperlipidemia was only found in 21 patients (17.5%) with normal coronary arteries whereas it was found in 109 (28.2%) patients with atherosclerotic CAD (p = 0.019).

Simple logistic regression (Table 2) shows that increase in age by one year increased the odds of atherosclerotic CAD by 11% (95%CI: 1.08-1.16, p <0.001). The odds of atherosclerotic CAD were higher in males (COR: 6.56 95%CI: 3.91-11.00, p <0.001) versus females and sub-continent nationals (COR: 10.77, 95%CI: 4.27-27.14, p <0.001). Smoking (COR: 2.93, 95%CI: 1.91-4.51, p <0.001), hyperlipidemia (COR: 1.85, 95%CI: 1.10-3.11, p: 0.021) and diabetes (COR: 1.98, 95%CI: 1.17-3.35, p=0.011) significantly increased the likelihood of atherosclerotic CAD.

Multiple regression analysis shows that increasing age, being male, sub-continent nationality, smoking and diabetes were significantly associated with increased likelihood of atherosclerotic CAD. See Table 3.

## Discussion

It is well established that smoking, diabetes, hypertension, increasing age, and male sex, are significant risk factors for CAD 12. This has also been demonstrated in our study.

Patients with premature coronary disease referred to coronary angiography commonly have less extensive coronary artery disease than older patients 13. Young patients with AMI are very frequently heavy smokers and have a high incidence of angiographically normal coronary arteries 14. Another study showed young men and women more often had angiographically normal coronary arteries compared to older patients 15.

AMI with normal coronary arteries is typically diagnosed in young patients. Most cases occur under 50 years of age 16-17. Age range between 19-29 years, and female sex were the only categories in which the number of patients with angiographically normal coronary arteries was higher than those with atherosclerotic CAD.

This could mean that patients with normal coronary anatomy may not be having an ACS and could be presenting with symptoms due to other conditions, or out of fear that they may be experiencing an ACS. In both categories of patients (with and without CAD), the number of patients increased in the 30-39 age range. However, while the number of patients having atherosclerotic CAD increased in the 40-50 age range; this was not the case in the group of patients with normal coronary arteries, in which there was a decrease in number. The reasons for this may have important implications for screening in the older population of risk factors for development of atherosclerotic coronary artery disease 18-21.

Ethnically, among patients with CAD, the majority were from the Subcontinent, a high percentage as compared to the other ethnic groups. This was not the case among patients with normal coronary arteries, as the most prevalent group were Emiratis. This may be due to the fact that those from the Subcontinent are at higher risk for CAD due to lifestyle habits and genetic predisposition 22-24, and Arabs and Emiratis may be more cautious about their symptoms which results in them coming to the hospital but not having any abnormal angiographic finding.

Family history was a risk factor in which both groups were comparable. In one study a positive family history of premature coronary disease was significantly more prevalent only in young men 15. Family history has a part to play in risk of development of CAD but it may not be a strong risk factor 25-26.

A factor that is pivotal, nevertheless, is a history of smoking. As “smoking” in this study was not limited to cigarettes, this highlights the importance of a need for robust smoking cessation programs as well as smoking prevention programs, possibly to be implemented in schools around the UAE, to educate the public about the dangers of smoking not only cigarettes but other sources, as many of the public are not aware of the true threat of these other forms 27-28. In the United Arab Emirates, around 15.7% adult males and 2.4% adult females are current tobacco smokers 29.

In analyzing BMI among the different categories, it was found that in both instances, the highest percentage of patients belonged to the “overweight” classification. In both groups there was a decrease in the number of patients when comparing the “overweight” category to the “obese”. This decrease was more prominent in CAD patients. This may be due to the increased likelihood of atypical symptoms in obese patients 30-31.

One study observed that high plasma triglyceride and low HDL cholesterol levels are associated with premature coronary artery disease 13. In our analysis, TG level was significantly higher in the CAD group.

There was no significant difference in the total cholesterol, LDL, and HDL levels between the patients with atherosclerotic coronary artery disease and normal coronary arteries. Atherosclerosis is an event that starts taking place in childhood 32. In the present era more than ever, high cholesterol in the general population has reached epidemic levels. 33-34. This may be the reason why there is no difference between those with normal vs abnormal coronary arteries**.**

However, as expected, being that hyperlipidemia one of the most important risk factors for CAD 35-36, there was a significant difference (p = 0.019) between the two groups. The risks associated with high blood cholesterol can be reduced by screening and early intervention. Current clinical practice guidelines provide evidenced-based standards for detection, treatment, and control of high blood cholesterol 37.

Diabetes is a known major risk factor for development of atherosclerotic CAD 38, and this was reflected in our cohort (p = 0.01).Prevalence of hypertension in the cohort was comparable between the two groups of patients. Hypertension as a risk factor in development of CAD has been well studied 12,39-40 and the most common end result of hypertension is CAD 41. Hypertension predisposes to all clinical manifestations of CHD including myocardial infarction, angina pectoris, and sudden death. Even high normal BP values are associated with an increased risk of CVD 42-43.

Risk factors notwithstanding, angiographically occult CAD has been described as a potential mechanism for coronary events in patients with ACS and normal or near-normal findings on cardiac angiography. Consideration also should be given to other causes such as angiograms that are inadequate or misinterpreted by visual analysis. No matter how precisely measured, the angiogram of a complex lesion poorly represents the real lumen size 44.

Other potential causes include myocardial infarction caused by coronary spasm 45, microembolization 46, and a misdiagnosis of ACS in patients with a variety of different clinical entities, such as myocarditis and Takatsubo cardiopathy, 47, among others. CMRI may be used to rule out potential causes, and is a useful tool for the management of ACS presenting with normal coronary angiography, as it aids diagnosis and adaptation of treatment in a large proportion of cases 48-50. The prognosis of patients with ACS and normal coronary arteries was considered to be excellent, based off of several small studies showing a low recurrence rate of ACS and a high 10-year survival rate 16. However, a more recent study that included 9796 patients with ACS undergoing coronary angiography showed less favorable outcomes 17 as well as in one study that assessed myocardial injury in ACS with normal coronary angiography using CMRI 48. Patients presenting with typical symptoms of ACS but without critical obstruction on visual angiography have a prognosis that is not as benign as previously thought 51, making it all the more important to find a way to risk stratify this subset of patients.

Limitations of our study include its retrospective and cross sectional nature. Final outcomes were not evaluated as a part of this study Additionally, non-obstructive stenosis may possibly be underestimated by angiography.

## Conclusion

Risk factors were present significantly more in the CAD group. No particular risk factor stood out for the development of ACS in those with normal coronary arteries, other than mean BMI being slightly higher in this group. Based on our analysis, no single variable can accurately predict the risk for ACS in patients with no or minimal CAD risk factors and normal coronaries by coronary angiography (CAG). Angiographically occult CAD has been described as one of many potential causes, however, larger studies are needed and other etiologies of chest pain and positive troponin such as myocarditis or coronary spasm, should be entertained. Treatment of these cases differ and would require correct diagnosis and further investigations such as cardiac MRI prior to initiation of long-term treatment, which can have lifelong implications on these young patients.

To our knowledge, very few studies have been done in the young population with angiographically normal coronary arteries to determine possible risk factors for development of ACS 52. Further research needs to be done to determine whether the risk factors that were common amongst both groups are coincidental, or a cause of ACS in those with normal coronary arteries.

## Figures and Tables

**Figure 1:**
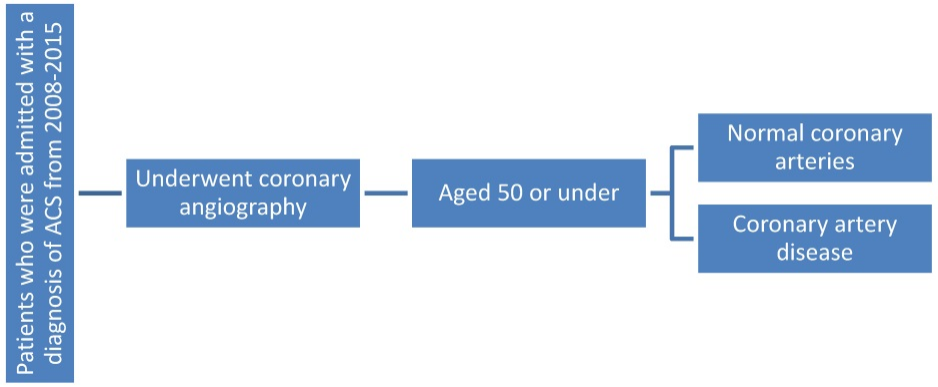
Flowchart showing patient selection process.

**Table 1:** Risk factors of atherosclerotic CAD

Variables	N	All, n (%)	Normal coronary arteries, n (%)	Atherosclerotic CAD, n (%)	*P-*Value
All		593	126 (20.1)	467 (79.9)	
Age - years,	592	37.9± 5.3	35.3± 6.8	38.7± 4.6	<0.001*
**Age categories**					<0.001*
19-29 years	592	46 (7.8)	26 (20.6)	20 (4.3)	
30-39 years		253 (42.7)	55 (43.7)	198 (42.5)	
40-50 years		293 (49.5)	45 (35.7)	248 (53.2)	
**Gender**					<0.001*
Female	593	73 (12.3)	41 (32.5)	32 (6.9)	
Male		520 (87.7)	85 (67.5)	435 (93.1)	
**Ethnicity**					<0.001*
Arabic	520	102 (19.6)	36 (30.0)	66 (16.5)	
Emirati		117 (22.5)	38 (31.7)	79 (19.7)	
Sub-continent		279 (53.7)	33 (27.5)	246 (61.5)	
Other		22 (4.2)	13 (10.8)	9 (2.3)	
Family history of CHD	450	112 (24.9)	25 (22.7)	87 (25.6)	0.546
Smoker	495	283 (57.2)	43 (37.1)	240 (63.3)	<0.001*
BMI - kg/m^2^,	475	28.1 ± 6.4	29.7 ±8.2	27.5±5.6	0.008*
**BMI**					0.073
Normal	475	159 (33.5)	33 (28.5)	126 (35.1)	
Overweight		194 (40.8)	44 (37.9)	150 (41.8)	
Obese		122 (25.7)	39 (33.6)	83 (23.1)	
TC (mmol/L)	392	4.7 ±1.4	4.5± 1.1	4.8 ± 1.5	0.108
LDL (mmol/L)	391	3.1 ± 1.3	2.9 ± 1.0	3.2±1.3	0.114
HDL (mmol/L)	393	0.8 ± 0.2	0.9 ± 0.3	0.8 ± 0.2	0.056
TG (mmol/L)	392	1.6 ± 0.9	1.4± 1.0	1.7 ± 1.0	0.047*
Hyperlipidemia	507	130 (25.6)	21 (17.5)	109 (28.2)	0.019*
Diabetes	508	130 (25.6)	20 (16.7)	110 (28.3)	0.010*
Hypertension	508	174 (34.3)	38 (31.7)	136 (35.1)	0.495

TC: Total Cholesterol; LDL: low-density lipoprotein; HDL: high-density lipoprotein; TG: Triglycerides; CHD: Coronary Heart Disease.

**Table 2:** Simple logistic regression analysis of Atherosclerotic CAD with selected variables

Variables	Atherosclerotic CAD	
	COR (95% CI)	*P-*Value
Ages	1.12 (1.08-1.16)	<0.001*
**Sex**		
Female	1	
Male	6.56 (3.91-11.00)	<0.001*
**Ethnicity**		
Arab	2.65 (1.03- 6.79)	0.043*
Emirati	3.00 (1.18- 7.64)	0.021*
Sub-continent	10.77 (4.27-27.14)	<0.001*
Other	1	
Family history of CHD	1.17 (0.70-1.94)	0.547
Smoker	2.93 (1.91-4.51)	<0.001*
BMI - kg/m^2^	0.95 (0.92- 0.98)	0.002*
Hyperlipidemia	1.85 (1.10-3.11)	0.021*
Diabetes	1.98 (1.17-3.35)	0.011*
Hypertension	1.16 (0.75-1.80)	0.495

COR: Crude Odds Ratio, CI: Confidence Interval, SD: Standard Deviation, CHD: Coronary Heart Disease, BMI: Body Mass Index.

**Table 3:** Multiple logistic regression analysis of Atherosclerotic CAD with selected variables

Variables	Atherosclerotic CAD	
	AOR (95% CI)	*P*-Value
Age - years	1.12 (1.07-1.17)	<0.001*
**Sex**		
Female	1	
Male	5.07 (2.22-10.66)	<0.001*
**Ethnicity**		
Arab	1.87 (0.58-6.01)	0.293
Emirati	1.83 (0.57-5.83)	0.309
Sub-continent	5.98 (1.89-18.76)	0.002*
Other	1	
Smoker	2.05 (1.20-3.50)	0.009*
BMI - kg/m^2^	0.99 (0.95- 1.03)	0.634
Hyperlipidemia	1.98 (0.99-3.96)	0.054
Diabetes	2.03 (0.99-4.14)	<0.052

AOR: Adjusted Odds Ratio, CI: Confidence Interval, SD: Standard Deviation, BMI: Body Mass Index.
